# Secrecy Capacity of a Class of Erasure Wiretap Channels in WBAN

**DOI:** 10.3390/s18124135

**Published:** 2018-11-26

**Authors:** Bin Wang, Jun Deng, Yanjing Sun, Wangmei Guo, Guiguo Feng

**Affiliations:** 1 School of Communication Engineering, Xi’an University of Science and Technology, Xi’an 710054, China; wangbin@mail.xidian.edu.cn (B.W.); yanjingsun_cn@163.com (Y.S.); 2 School of Information and Control Engineering, China University of Mining and Technology, Xuzhou 221116, China; 3 State Key Laboratory of Integrated Services Networks, Xidian University, Xi’an 10071, China; wangmeiguo@xidian.edu.cn (W.G.); fengguiguo@163.com (G.F.)

**Keywords:** wiretap channel II, secrecy capacity, finite state Markov erasure wiretap channel, WBAN

## Abstract

In wireless body area networks (WBANs), the secrecy of personal health information is vulnerable to attacks due to the openness of wireless communication. In this paper, we study the security problem of WBANs, where there exists an attacker or eavesdropper who is able to observe data from part of sensors. The legitimate communication within the WBAN is modeled as a discrete memoryless channel (DMC) by establishing the secrecy capacity of a class of finite state Markov erasure wiretap channels. Meanwhile, the tapping of the eavesdropper is modeled as a finite-state Markov erasure channel (FSMEC). A pair of encoder and decoder are devised to make the eavesdropper have no knowledge of the source message, and enable the receiver to recover the source message with a small decoding error. It is proved that the secrecy capacity can be achieved by migrating the coding scheme for wiretap channel II with the noisy main channel. This method provides a new idea solving the secure problem of the internet of things (IoT).

## 1. Introduction

Due to the openness of wireless communication, the personal health information, which is exchanged on the wireless channel in WBAN, is readily fetched and attacked by hackers. To address this issue, there are usually two ways to enhance the security of wireless communications: one is the security guaranteed by information theory in Refs. [1,2,3], another is the security verified by the computational complexity in Refs. [4,5]. In this paper, we aim to study the secure transmission problem in WBAN on the basis of the information theory. Here, the secure transmission indicates the way to code the transmitted data so that the attackers cannot get the data. The concept of wiretap channel is introduced by Wyner in Ref. [6]. In his model, the source message was sent to the targeted user via a discrete memoryless channel (DMC). Meanwhile, an eavesdropper was able to tap the transmitted data via a second DMC. It was supposed that the eavesdropper knew the encoding scheme and decoding scheme. The object was to find a pair of encoder and decoder such that the eavesdropper’s level of confusion on the source message was as high as possible, while the receiver could recover the transmitted data with a small decoding error. Wyner’s wiretap channel model is called the discrete memoryless wiretap channel, since the main channel output was taken as the input of the wiretap channel in Ref. [7].

After Wyner’s pioneering work, the models of wiretap channels have been studied from various aspects. Csiszar and Korner considered a more general wiretap channel model called the broadcast channels with confidential messages (BCCs) in Ref. [8]. The wiretap channel was not necessarily a degraded version of the main channel. Moreover, they also considered the case where public data was supposed to be broadcasted through both main channel and wiretap channel. The degraded wiretap channels with discrete memoryless side information accessed by the encoder were considered in Refs. [9,10,11]. BCCs with causal side information were studied in Ref. [12]. Communication models with channel states known at the receiver were considered in Refs. [13,14]. Ozarow and Wyner considered another wiretap channel model called wiretap channel of type II [15]. The secrecy capacity was established there. In that model, the source data was encoded into *N* digital symbols and transmitted to the targeted user through a binary noiseless channel. Meanwhile, the eavesdropper was able to observe an arbitrary μ-subcollection of those symbols.

In the last few decades, a lot of capacity problems related to the wiretap channel II were studied. A special class of non-DMC wiretap channel was studied in Ref. [16]. The main channel was a DMC instead of noiseless, and the eavesdropper observed μ<N digital symbols through a uniform distribution. An extension of wiretap channel II was studied in Ref. [17], where the main channel was a DMC and the eavesdropper was able to observe μ digital bits through arbitrary strategies.

The model of finite-state Markov channel was first introduced by Gilbert [18] and Elliott [19]. They studied a kind of Markov channel model with two states, which is known as the Gilbert–Elliott channel. In their channel model, one state was related to a noiseless channel and the other state was related to a totally noisy channel. Wang in Ref. [20] extended the Gilbert–Elliott channel and considered the case with finite states.

**Figure 1:**
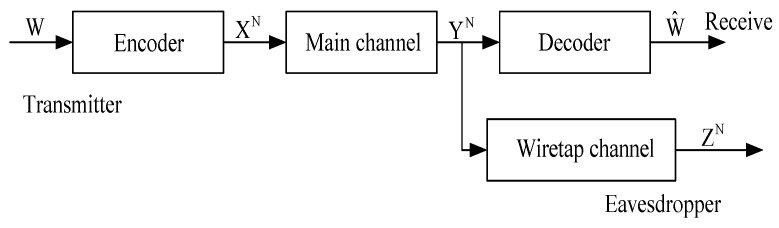
Communication model of degraded wiretap channels.

This paper discusses finite-state Markov erasure wiretap channel (FSME-WTC) (see Figure 1). In this new model, the source data *W* is encoded into *N* digital symbols, denoted by $X^{N}$, and transmitted to the targeted user through a DMC. The eavesdropper is able to observe the transmitted symbols through a finite-state erasure Markov channel (FSMEC). Secrecy capacity of this new communication model is established, based on the coding scheme devised by the authors in Ref. [17].

The model of FSME-WTC can be applied to model the security problem of WBAN readily. Let us suppose that there are N sensors in WBAN. Then, we can treat the collection of symbols obtained from the sensors as a digital sequence of length N transimitted over an imaginary channel. The imaginary channel is not DMC because the symbols from the sensors are correlated. Markov chain is an important model to characterize the correlation of random variables since it will not bring too much complexity of the system. The wiretap channel is set as an erasure channel to model the situation where the attacker in WBAN is able to tap data from only part of the sensors. Thus, our model of FSME-WTC is to ensure that the attacker is not able to get any information from the WBAN when he/she can only observe data from at most Nα sensors.

The importance of this model is obvious. As the technology of 5G advances towards the stage of commercial applications, wireless networks are becoming more and more significant in our daily lives [21,22]. Therefore, the security problem of wireless communication is critical from the aspects of both theory and engineering. Meanwhile, the finite state Markov channel is a common model to character the properties of wireless communication. Hence, the results of this paper are meaningful to many kinds of wireless networks with high confidentiality requirements, such as WBAN and IoT.

The remainder of this paper is organized as follows. The formal statements of Finite-state Markov Erasure Wiretap Channel and the capacity results are given in Section 2 (see also Figure 1). The secrecy capacity of this model is established in Theorem 1. Some concrete examples of this communication model are given in Section 3. The converse part of Theorem 1, relying on Fano’s inequality and Proposition 1, is proved in Section 4. The direct part of Theorem 1, based on Theorem 1 in [17], is proved in Section 5. Section 6 gives the proof of Proposition 1, and Section 7 finally concludes this paper.

## 2. Notations, Definitions and the Main Results

Throughout this paper, N is the set of positive integers. [1:N]={1,2,…,N} is the set of positive integers no greater than *N* for any N∈N. For any index set I⊆[1:N] and random vector $Y^{N}=\left(Y_{1},Y_{2},\ldots ,Y_{N}\right)$, denote by $Y_{I}^{N}=\left(Y_{1}^{\prime },Y_{2}^{\prime },\ldots ,Y_{N}^{\prime }\right)$ the “projection” of $Y^{N}$ onto the index set I such that $Y_{i}=Y_{i}^{\prime }$ for all i∈I, and $Y_{i}=?$, otherwise.

Let Y be any finite alphabet not containing the “error” letter ? and $Y_{I}^{N}=\left\{\left(y_{1},y_{2},\ldots ,y_{N}\right):y_{i}\in Y\;\mathrm{for}\;i\in I,\;\mathrm{and}\;i=?\;\mathrm{for}\;i\notin I\right\}$. It follows that $Y_{I}^{N}$ is distributed on $Y_{I}^{N}$ for any random vector $Y^{N}$ over $Y^{N}$.

**Example** **1.** *Let N=5, I=1,3 and X=0,1. Then,*

$$X_{I}^{N}=\left\{\left(0?0??\right),\left(0?1??\right),\left(1?0??\right),\left(1?1??\right)\right\}.$$

*Let $X^{N}=\left(X_{1},X_{2},X_{3},X_{4},X_{5}\right)$ be an arbitrary random vector distributed on $X^{N}$. Then, the random vector $X_{I}^{N}=\left(X_{1},?,X_{3},?,?\right)$ is distributed on $X_{I}^{N}$.*

**Definition** **1.** **(Encoder)** *Let the source message W be uniformly distributed on a certain message set W. The (stochastic) encoder $q_{E}$ is specified by a matrix of conditional probability $q_{E}\left(x^{N}|w\right)$ with $x^{N}\in X^{N}$ and w∈W. The value of $q_{E}\left(x^{N}|w\right)$ specifies the probability that we encode message w encoded into the sequence $x^{N}$.*

**Definition** **2.** **(Main** **channel)** *The main channel is a DMC, whose input alphabet is X and output alphabet is Y, where ?∉X∪Y. The transition probability matrix of the main channel is denoted by $Q_{MC}\left(y|x\right)$ with x∈X and y∈Y. The input and output of the main channel are denoted by $X^{N}$ and $Y^{N}$, respectively. For any $x^{N}\in X^{N}$ and $y^{N}\in Y^{N}$, it follows that*

$$Pr\left\{X^{N}=x^{N},Y^{N}=y^{N}\right\}=Pr\left\{X^{N}=x^{N}\right\}Q_{M}C\left(y^{N}|x^{N}\right),$$

*where*

$$\begin{array}{c} Q_{MC}\left(y^{N}|x^{N}\right)=\prod_{i=1}^{N}Q_{MC}\left(y_{i}|x_{i}\right). \end{array}$$

**Remark** **1.** *From the property of DMC, it holds that*

$$\begin{array}{c} H\left(Y^{N}|X^{N}\right)=\sum_{i=1}^{N}H\left(Y_{i}|X_{i}\right). \end{array}$$

**Definition** **3.** **(Wiretap** **channel)** *Let $T_{n},n\in N$ be the channel state of FSMEC at time n satisfying that $T_{1}\to T_{2}\to \ldots \to T_{N}\to \ldots$ forms a Markov chain. The transition of channel states is homogeneous, i.e., the conditional probability $Pr\{T_{n}=t_{n}|T_{n-1}=t_{n-1}\}$ is independent from the time index n. Moreover, the channel states are stationary, i.e., $T_{1},T_{2},\ldots ,T_{N},\ldots$ share a generic probability distribution $p_{T}$ on a common finite set T of channel states. Moreover, let $Q_{T}\left(t^{\prime }|t\right)$ be the probability that the state at the next time slot is changed to $t^{\prime }$ when the state is t currently. It follows that*

$$\begin{array}{c} Pr\left\{T^{N}=t^{N}\right\}=p_{T}\left(t_{1}\right)\cdot \prod_{i=2}^{N}Q_{T}\left(t_{i}|t_{i}-1\right) \end{array}$$

*for $t^{N}\in T^{N}$. The input of FSMEC is a digital sequence $Y^{N}$, which is actually the main channel output. Denote by $Z^{N}$ the wiretap channel output. For each time slot n, the channel is either totally noisy, i.e., $Z_{n}=?$ or totally noiseless, i.e., $Z_{n}=Y_{n}$, which depends on the value of $T_{n}$. Thus, the channel output $Z_{n}$ is totally determined by the channel input $Y_{n}$ and the channel state $T_{n}$. Let $T_{1}$ be the set of states under which the channel is noiseless. Then, it follows that $T_{0}=T-T_{1}$ contains the states where the channel is totally noisy. Denote by $Q_{WC}\left(z|y,t\right)$ the probability that the channel outputs z when the channel input is y and the channel state is t. It follows that*

$$\begin{array}{c} Q_{WC}\left(z|y,t\right)=\left\{\begin{array}{c} \delta \left(z,y\right)t\in T_{1},\\ \delta \left(z,?\right),t\in T_{0}, \end{array}\right. \end{array}$$

*where*

$$\begin{array}{c} \delta \left(a,b\right)=\left\{\begin{array}{c} 1,a=b,\\ 0,a\neq b. \end{array}\right. \end{array}$$

*For any $y^{N}\in Y^{N}$, $z^{N}\in Z^{N}$ and $t^{N}\in T^{N}$, it is readily obtained that*

$$\begin{array}{c} Pr\left\{Y^{N}=y^{N},Z^{N}=z^{N}|T^{N}=t^{N}\right\}=Pr\left\{Y^{N}=y^{N}\right\}\prod_{i=1}^{N}Q_{WC}\left(z_{i}|y_{i},t_{i}\right). \end{array}$$

**Remark** **2.** *Throughout this paper, it is supposed that $T^{N}$ is independent from W, $X^{N}$ and $Y^{N}$.*

**Proposition** **1.** *$X^{n}\to Z^{n}\to T^{n}$ forms a Markov chain for every 1≤n≤N.*

**Proof.**  The proof of Proposition 1 is given in Section 6. Proposition 1 would be used to establish the converse part of Theorem 1 (see Section 4). □

**Definition** **4.** **(Decoder)** *The decoder is specified by a mapping $f_{D}:Y^{N}\to W$. To be particular, the estimation of the source message is $\hat{W}=Y^{N}$, where $Y^{N}$ is the main channel output. The average decoding error probability is denoted by $P_{e}=Pr\left\{W\neq \hat{W}\right\}$.*

**Definition** **5.** **(Achievability)** *A positive real number R is said to be achievable, if, for any real number ε>0, one can find an integer $N_{0}$ such that, for any $N>N_{0}$, there exists a pair of encoder and decoder of length of length N satisfying that*

(1) $$\begin{array}{c} \frac{1}{N}log\left|W\right|>R-\varepsilon ,\frac{1}{N}I\left(W;Y^{N}\right)<\varepsilon \;and\;P_{e}<\varepsilon . \end{array}$$

**Definition** **6.** **(Secrecy** **capacity)** *A real number $C_{s}$ is said to be the secrecy capacity of the communication model if it is achievable for every $0\leq R\leq C_{s}$ and unachievable for every $R>C_{s}$.*

**Theorem** **1.** *Let $B_{n}$ be the function of $T_{n}$ defined in Definition 3 such that $B_{n}=1$ if $T_{n}\in T_{1}$, and $B_{n}=0$, otherwise. If it follows that*

(2) $$\begin{array}{c} \lim_{N\to \infty }Pr\{|\frac{1}{N}\sum_{n=1}^{N}B_{n}-\alpha \left|<\iota \right\}=1 \end{array}$$

*for any ι>0, the secrecy capacity of the communication model in Figure 1 is $\left(1-\alpha \right)C_{M}$, where $C_{M}$ is the capacity of the main channel, i.e.,*

(3) $$\begin{array}{c} C_{M}=\max_{P_{X}}I\left(X;Y\right). \end{array}$$

**Proof.**  The proof of Theorem 1 is divided into the following two parts. The first part, given in Section 4, proves that every achievable real number *R* must satisfy $R\leq \left(1-\alpha \right)C_{M}$, which is the converse half of the theorem. The second part, given in Section 5, proves that every real number *R* satisfying $0\leq R\leq \left(1-\alpha \right)C_{M}$ is achievable, which is the direct half. □

Theorem 1 claims that, if the Markov chain $\left\{T_{n}\right\}$ satisfies Label (2), then the secrecy capacity of the wiretap channel model depicted in Figure 1 is $\left(1-\alpha \right)C_{M}$. In the rest of this section, we will introduce a class of Markov chains satisfying (2) in Theorem 2, and provide the secrecy capacity of the related wiretap channel model in Corollary 1.

A stationary Markov chain is call ergodic if, for each pair of states $t,t^{\prime }\in T$, it is possible to go from state *t* to $t^{\prime }$ in expected finite steps. One can prove that, if a Markov of chain is ergodic, the stationary probability distribution of the state is unique.

**Theorem** **2.** **(Law** **of** **Large** **Number** **for** **Markov** **Chain)** *If the Markov chain $\left\{T_{n}\right\}$ is ergodic, let π be the unique stationary distribution of the state. Then, it follows that*

$$\begin{array}{c} \lim_{N\to \infty }\frac{1}{N}\sum_{i=1}^{N}I\left(T_{n}=t\right)=\pi \left(t\right) \end{array}$$

*for each channel state t, where $I(T_{n}=t)$ is* 1 *or* 0*, indicating whether $T_{n}=t$ is true or not.*

With the theorem above, we immediately obtain that

**Corollary** **1.** *If the Markov chain $\left\{T_{n}\right\}$ is ergodic with the unique stationary distribution π over T, then the secrecy capacity of the wiretap channel model depicted in Figure 1 is given by*

$$\begin{array}{c} C_{s}=\left(1-\pi \left(T_{1}\right)\right)C_{M}, \end{array}$$

*where $C_{M}$ is the capacity of the main channel, and*

$$\begin{array}{c} \pi \left(T_{1}\right)=\sum_{t\in T_{1}}\pi \left(t\right). \end{array}$$

## 3. Examples

This section gives two simple examples of FSMEC defined in Definition 3. Example 2 is for discrete memoryless erasure channel (DMEC) and Example 3 is for a simple two-state FSMEC.

**Example** **2.** *Suppose that the set of channel states T=0,1 with $T_{1}=1$ and $T_{0}=0$. Meanwhile, let*

(4) $$\begin{array}{c} p_{T}\left(0\right)=Q_{t}\left(0|0\right)=Q_{t}\left(0|1\right)=1-\alpha \end{array}$$

*and*

$$p_{T}\left(1\right)=Q_{t}\left(1|0\right)=Q_{t}\left(1|1\right)=\alpha .$$

**Figure 2:**
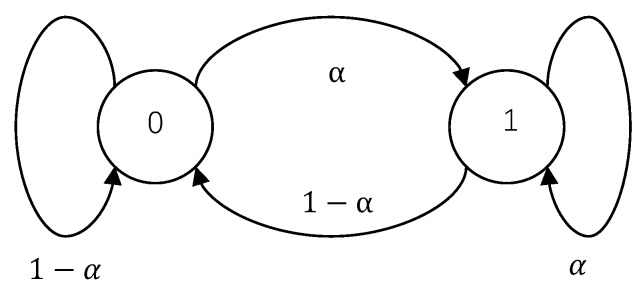
State transition diagram of discrete memoryless erasure channels.

*The state transition diagram of the channel states in this example is depicted in Figure 2. It is obvious that the FSMEC is in fact specialized into a DMEC with the transition probability*

$$Q_{WC}\left(z|y\right)=\left\{\begin{array}{cc} \alpha ,\; & z=y,\\ 1-\alpha ,\; & z=?,\\ 0,\; & otherwise. \end{array}\right.$$

*From Theorem 2 in Ref. [6], the secrecy capacity of the communication model in Figure 1, with DMEC as the wiretap channel, is*

$$\begin{array}{c} \max_{P_{X}}I\left(X:Y|Z\right)\\ \;=\max_{P_{X}}I\left(X;Y\right)-I\left(X;Z\right)\\ \;\overset{\left(a\right)}{=}\max_{P_{X}}I\left(X;Y|T\right)-I\left(X;Z|T\right)\\ \;=\max_{P_{X}}\left\{\left[I\left(X;Y|T=0\right)-I\left(X;Z|T=0\right)\right]P_{T}\left(0\right)+\left[I\left(X;Y|T=1\right)-I\left(X;Z|T=1\right)\right]P_{T}\left(1\right)\right\}\\ \;\overset{\left(b\right)}{=}\max_{P_{X}}I\left(X;Y|T=0\right)P_{T}\left(0\right)\\ \;\overset{\left(c\right)}{=}\max_{P_{X}}I\left(X;Y\right)P_{T}\left(0\right)\\ \;\overset{\left(d\right)}{=}\left(1-\alpha \right)C_{M}, \end{array}$$

*where X and Y are the input and output of the main channel, respectively, and Z is the output of the wiretap channel under the channel state T; (a) follows from the facts that X→Z→T forms a Markov chain (cf. Proposition 1) and T is independent from X and Y; (b) follows from the fact that Y=Z when T=1, and Z is determined when T=0; (c) follows from the assumption that T is independent from X and Y; and (d) follows from (3) and (4).*

*Clearly, Formula (2) holds with $B_{n}=T_{n}$. Thus, in this case, the result of Theorem 1 in this paper coincides with that of Theorem 2 in Ref. [6].*

**Example** **3.** *Let $T=0,1,T_{1}=1,T_{0}=0,$*

$$p_{T}\left(0\right)=p_{T}\left(1\right)=\frac{1}{2},$$

$$Q_{t}\left(0|0\right)=Q_{t}\left(1|1\right)=p,$$

$$Q_{t}\left(1|0\right)=Q_{t}\left(0|1\right)=1-p,$$

**Figure 3:**
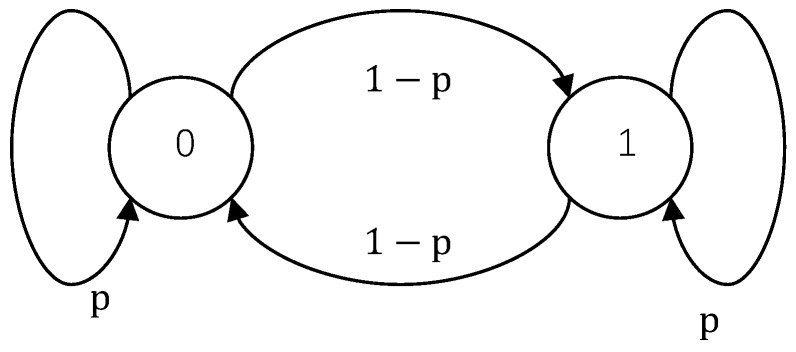
State transition diagram of a two-state Markov chain.

*and $B_{n}=T_{n}$. We arrive at a simple two-state Markov erasure channel whose transition diagram is depicted in Figure 3. Furthermore, observe that*

$$\begin{array}{ll} D\left[\sum_{n=1}^{N}B_{n}\right] & =D\left[\sum_{n=1}^{N}T_{n}\right]\\ & =E\left[{\left(\sum_{n=1}^{N}T_{n}\right)}^{2}\right]-{\left(E\left[\sum_{n=1}^{N}T_{n}\right]\right)}^{2}\\ & =\left(\sum_{n=1}^{N}\sum_{m=1}^{N}E\left[T_{n}T_{m}\right]\right)-\frac{N^{2}}{4}\\ & =\sum_{n=1}^{N}\frac{\left(N-n\right)}{2}{\left(2p-1\right)}^{n}, \end{array}$$

*where the last equality follows because $E\left[T_{n}T_{m}\right]=\frac{1}{2}$ when m=n, and*

$$\begin{array}{ll} E[T_{n}T_{m}] & =Pr\{T_{m}=1,T_{n}=1\}\\ & =Pr\left\{T_{m}=1,T_{n-1}=1,T_{n}=1\right\}+Pr\left\{T_{m}=1,T_{n-1}=0,T_{n}=1\right\}\\ & =Pr\left\{T_{m}=1,T_{n-1}=1\right\}Q_{T}\left(1|1\right)+Pr\left\{T_{m}=1,T_{n-1}=0\right\}Q_{T}\left(1|0\right)\\ & =E\left[T_{m}T_{n-1}\right]p+\left(\frac{1}{2}-E\left[T_{m}T_{n-1}\right]\right)\left(1-p\right)\\ & =\left(2p-1\right)E\left[T_{m}T_{n-1}\right]+\frac{1-p}{2}\\ & =\ldots \\ & ={\left(2p-1\right)}^{n-m}E\left[T_{m}T_{m}\right]+\frac{1-p}{2}\sum_{i=1}^{n-m-1}{\left(2p-1\right)}^{i}\\ & =\frac{1+{\left(2p-1\right)}^{n-m}}{4} \end{array}$$

*when m<n. It is obvious that*

$$\lim_{N\to \infty }\frac{1}{N^{2}}D\left[\sum_{n=1}^{n}B_{n}\right]=0$$

**Figure 4:**
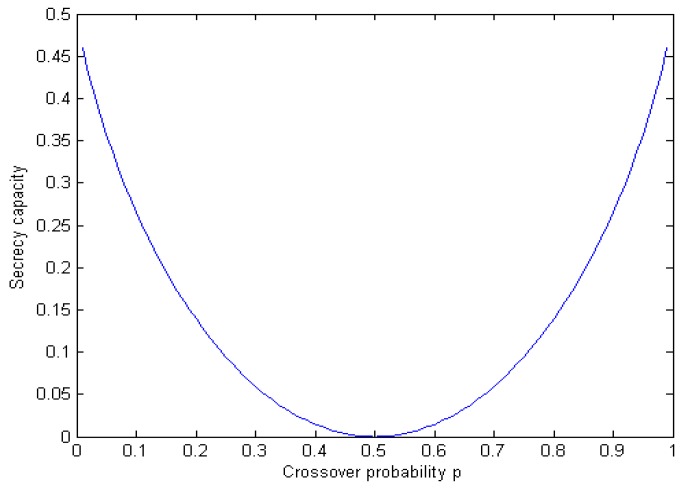
Secrecy capacity of the two-state Markov erasure wiretap channel in Example 3.

*for 0<p<1. Formula (2) is then established immediately from the Markov Large Number Law. Applying Theorem 1, the secrecy capacity of the communication model in this case is $\frac{1}{2}C\left(p\right)$. Figure 4 shows the relationship between the secrecy capacity and the crossover probability p in this example.*

## 4. Converse Half of Theorem 1

This section proves that every achievable real number *R* must satisfy $R\leq \left(1-\alpha \right)C_{M}$. The proof is based on Fano’s inequality (cf. Formula (76) in Ref. [6]) and Proposition 1.

For any give ι>0 and ε>0, Formula (2) indicates that

$$Pr\{\frac{1}{N}\sum_{n=}^{N}B_{n}>\alpha -\iota \}>1-\varepsilon$$

or equivalently

(5) $$\begin{array}{c} Pr\{|I\left(T^{N}\right)|>N\left(\alpha -\iota \right)\}>1-\varepsilon \end{array}$$

when *N* is sufficiently large, where

$$I\left(t^{N}\right)=\left\{n\in \left[1:N\right]:t_{n}\in T_{1}\right\}.$$

Suppose that there exists a code of length *N* satisfying (1), i.e.,

$$\frac{1}{N}\log\left|W\right|>R-\varepsilon ,\frac{1}{N}I\left(W;Z^{N}\right)<\varepsilon \;\mathrm{and}\;P_{e}<\varepsilon .$$

Then, we have

$$\begin{array}{c} NR<\log|W|+N\varepsilon =H\left(W\right)+N\varepsilon =I\left(W;Y^{N}\right)+H\left(W|Y^{N}\right)+N\varepsilon <I\left(W;Y^{N}\right)+N\delta \left(P_{e}\right)+N\varepsilon , \end{array}$$

where $\delta (P_{e})\to 0$ as $P_{e}\to 0$, and the last inequality follows from the Fano’s inequality. Since $I(W;Z^{N})<N\varepsilon$, the formula above indicates that

(6) $$\begin{array}{c} NR<I\left(W;Y^{N}\right)-I\left(W;Z^{N}\right)+N\delta \left(P_{e}\right)+2N\varepsilon . \end{array}$$

The value of $I\left(W;Y^{N}\right)-I\left(W;Z^{N}\right)$ is upper bounded by

(7) $$\begin{array}{l} I\left(W;Y^{N}\right)-I\left(W;Z^{N}\right)\\ \;\overset{\left(a\right)}{=}I\left(W;Y^{N}|Z^{N}\right)\\ \;\overset{\left(b\right)}{\leq }I\left(X^{N};Y^{N}|Z^{N}\right)\\ \;=I\left(X^{N};Y^{N}\right)-I\left(X^{N};Z^{N}\right)\\ \;\overset{\left(c\right)}{=}I\left(X^{N};Y^{N}|T^{N}\right)-I\left(X^{N};Z^{N}|T^{N}\right), \end{array}$$

where (a) and (b) follow from the fact that $W\to X^{N}\to Y^{N}\to Z^{N}$ forms a Markov chain, and (c) follows from Proposition 1 and the fact that $T^{N}$ is independent from $X^{N}$ and $Y^{N}$.

For any $t^{N}\in T^{N}$, denoting $Z^{N}\left(t^{N}\right)=Y_{I(t^{N})}^{N}$, Formula (7) is further deduced by

(8) $$\begin{array}{l} I\left(W;Y^{N}\right)-I\left(W;Z^{N}\right)\\ \;\leq I\left(X^{N};Y^{N}|T^{N}\right)-I\left(X^{N};Z^{N}|T^{N}\right)\\ \;\overset{\left(a\right)}{=}I\left(X^{N};Y^{N}|Z^{N},T^{N}\right)\\ \;=\sum_{t^{N}\in T^{N}}\left(I\left(X^{N};Y^{N}|Z^{N},T^{N}=t^{N}\right)\cdot Pr\left\{T^{N}=t^{N}\right\}\right)\\ \;=\sum_{t^{N}\in T^{N}}\left(I\left(X^{N};Y^{N}|Z^{N}\left(t^{N}\right),T^{N}=t^{N}\right)\cdot Pr\left\{T^{N}=t^{N}\right\}\right)\\ \;\overset{\left(b\right)}{=}\sum_{t^{N}\in T^{N}}I\left(X^{N};Y^{N}|Z^{N}\left(t^{N}\right)\right)\cdot Pr\left\{T^{N}=t^{N}\right\}, \end{array}$$

where (a) follows because $X^{N}\to Y^{N}\to Z^{N}$ forms a Markov chain when given $T^{N}$, and (b) follows because $X^{N},Y^{N}$ and $Z^{N}\left(t^{N}\right)=Y_{I(t^{N})}^{N}$ are independent from $T^{N}$. For any fixed $t^{N}\in T^{N}$, denote ${\tilde{Z}}^{N}=Z^{N}\left(t^{N}\right)$. On account of the chain rule, we have

(9) $$\begin{array}{c} H\left(Y^{N}\right)=\sum_{n=1}^{N}H\left(Y_{n}|Y^{n-1}\right), \end{array}$$

(10) $$\begin{array}{c} H\left({\tilde{Z}}^{N}\right)=\sum_{n=1}^{N}H\left({\tilde{Z}}_{n}|{\tilde{Z}}^{n-1}\right), \end{array}$$

and

(11) $$\begin{array}{cc} H({\tilde{Z}}^{N}|X^{N}) & =\sum_{n=1}^{N}H\left({\tilde{Z}}_{n}|{\tilde{Z}}^{n-1},X^{N}\right)\\ & \leq \sum_{n=1}^{N}H\left({\tilde{Z}}_{n}|X^{n}\right). \end{array}$$

Moreover, from the property of DMC, Remark 1 yields

(12) $$\begin{array}{c} H\left(Y^{N}|X^{N}\right)=\sum_{n=1}^{N}H\left(Y_{n}|X_{n}\right). \end{array}$$

Combining Formulas (9)–(12), it follows that

(13) $$\begin{array}{c} I\left(W;Y^{N}\right)-I\left(W;Z^{N}\left(t^{N}\right)\right)\leq \sum_{n=1}^{N}\left(H\left(Y_{n}|Y^{n-1}\right)-H\left({\tilde{Z}}_{n}|{\tilde{Z}}^{n-1}\right)-H\left(Y_{n}|X_{n}\right)+H\left({\tilde{Z}}^{n}|X_{n}\right)\right). \end{array}$$

Considering that ${\tilde{Z}}^{(}n-1)\to Y^{(}n-1)\to Y_{n}\to {\tilde{Z}}_{n}$ forms a Markov chain, we have

$$I\left(Y^{n-1};Y_{n}\right)\geq I\left({\tilde{Z}}^{n-1};{\tilde{Z}}_{n}\right)$$

or equivalently

$$H\left(Y_{n}\right)-H\left({\tilde{Z}}_{n}\right)\geq H\left(Y_{n}|Y^{n-1}\right)-H\left({\tilde{Z}}_{n}|{\tilde{Z}}^{n-1}\right).$$

Substituting the formula above into Formula (13), we have

(14) $$\begin{array}{c} I\left(W;Y^{N}\right)-I\left(W:Z^{N}\left(t^{N}\right)\right)\\ \;=\sum_{n=1}^{N}\left(H\left(Y_{n}\right)-H\left({\tilde{Z}}_{n}\right)-H\left(Y_{n}|X_{n}\right)+H\left({\tilde{Z}}_{n}|X_{n}\right)\right)\\ \;=\sum_{n=1}^{N}\left(I\left(X_{n};Y_{n}\right)-I\left(X_{n};{\tilde{Z}}_{n}\right)\right). \end{array}$$

Noticing that

$$I\left(X_{n};{\tilde{Z}}_{n}\right)=\left\{\begin{array}{c} 0,t_{n}\in T_{1},\\ I\left(X_{n};Y_{n}\right),t\in T_{0}. \end{array}\right.$$

Formula (14) is further deduced by

$$\begin{array}{c} I\left(X^{N};Y^{N}\right)-I\left(X^{N};Z^{N}\left(t^{N}\right)\right)\leq \sum_{n=1}^{N}I\left(X_{n};Y_{n}\right)-I\left(X_{n};{\tilde{Z}}_{n}\right)=\sum_{n\notin I(t^{N})}I\left(X_{n};Y_{n}\right)\leq (N-|I\left(t^{N}\right)\left|\right)C_{M}. \end{array}$$

Substituting the formula above with Formula (8) gives

$$\begin{array}{c} I\left(X^{N};Y^{N}\right)-I\left(X^{N};Z^{N}\right)\\ \;\leq \sum_{t^{N}\in T^{N}}I\left(X^{N};Y^{N}|Z^{N}\left(t^{N}\right)\right)\mathrm{Pr}\left\{T^{N}=t^{N}\right\}\\ \;\leq \sum_{t^{N}\in T^{N}}(N-|ℑ\left(t^{N}\right)\left|\right)C_{M}\mathrm{Pr}\left\{T^{N}=t^{N}\right\}\\ \;\leq \mathrm{Pr}\left\{ℑ\left(T^{N}\right)\geq N\left(\alpha -\iota \right)\right\}N\left(1-\alpha +\iota \right)C_{M}+\mathrm{Pr}\left\{ℑ\left(T^{N}\right)<N\left(\alpha -\iota \right)\right\}NC_{M}\\ \;\leq N\left(1-\alpha +\iota +2\varepsilon \right)C_{M}, \end{array}$$

where the last inequality follows from (5). Combining (6) and the formula above yields

$$R<1-\alpha +\iota +4\varepsilon +\delta (P_{e}).$$

R≤1−α is finally established by letting ι,ε and $P_{e}$ converge to 0. This completes the proof of converse half.

## 5. Direct Half of Theorem 1

This section proves that every real number R satisfying $0<R\leq \left(1-\alpha \right)C_{M}$ is achievable, which is the direct half of Theorem 1. It suffices to prove the achievability of $\left(1-\alpha \right)C_{M}$. More precisely, for any given ε>0, we need to prove the existence of the encoder–decoder pair $\left(q_{E},f_{D}\right)$ such that

$$\frac{1}{N}\log\left|W\right|>R-\varepsilon ,\frac{1}{N}I\left(W;Z^{N}\right)<\varepsilon \;\mathrm{and}\;P_{e}<\varepsilon .$$

The proof is based on the following theorem.

**Theorem** **3.** *(Theorem 1 in Ref. [17]). Let a real number $0<\alpha^{\prime }1$ be fixed and given. For any N∈N and $\mu =N\alpha^{\prime }$, denote*

$${ℑ}_{\mu }={ℑ}_{\mu }\left(N\right)=\left\{I\subseteq \left[1:N\right]:|I|=\mu \right\}.$$

*Then, for any real numbers $\varepsilon^{\prime }>0$ and $0<R<\left(1-\alpha^{\prime }\right)C_{M}$, one can construct a code of length N over the DMC defined in Definition 2 such that*

$$\frac{1}{N}\log\left|W\right|>R-\varepsilon^{\prime },\max_{I\in {ℑ}_{\mu }}I\left(W;Y_{I}^{N}\right)<\varepsilon^{\prime },P_{e}<\varepsilon^{\prime }$$

*when N is sufficiently large.*

**Proof.**  Let

$$\alpha^{\prime }=\alpha +\iota$$

and

$$R=\left(1-\alpha -2\iota \right)C_{M}<\left(1-\alpha^{\prime }\right)C_{M}$$

for a small ι>0. Suppose that $\left(q_{E},f_{D}\right)$ is a code of length *N* satisfying

$$\begin{array}{c} \frac{1}{N}\log\left|W\right|>R-\varepsilon^{\prime }>\left(1-\alpha -2\iota \right)C_{M}-\varepsilon^{\prime }\\ \max_{I\in {ℑ}_{\mu }}I\left(W;Y_{I}^{N}\right)<\varepsilon^{\prime }\;\mathrm{and}\;P_{e}<\varepsilon^{\prime }. \end{array}$$

Applying the code $\left(q_{E},f_{D}\right)$ to the communication model in Figure 1, it is already satisfied that

$$\frac{1}{N}\log\left|W\right|>\left(1-\alpha \right)C_{M}-\varepsilon \;\mathrm{and}\;P_{e}<\varepsilon ,$$

when $\varepsilon^{\prime }$ and ι are sufficiently small. To establish $\frac{1}{N}I\left(W;Z^{N}\right)<\varepsilon$, let the value of *N* be sufficiently large such that

(15) $$\begin{array}{c} Pr|I\left(T^{N}\right)|<N\left(\alpha +\iota \right)>1-\varepsilon^{\prime }. \end{array}$$

The value of $I(W;Z^{N})$ is upper bounded by

$$\begin{array}{c} I(W;Z^{N})\\ \;\overset{\left(a\right)}{\leq }\;I\left(W;Z^{N}|T^{N}\right)\\ \;=\sum_{t^{N}\in T^{N}}I\left(W;Z^{N}|T^{N}=t^{N}\right)\mathrm{Pr}\left\{T^{N}=t^{N}\right\}\\ \;=\sum_{t^{N}\in T^{N}}I\left(W;Y_{ℑ(t^{N})}^{N}|T^{N}=t^{N}\right)\mathrm{Pr}\left\{T^{N}=t^{N}\right\}\\ \;\overset{\left(b\right)}{=}\;\sum_{t^{N}\in T^{N}}I\left(W;Y_{ℑ(t^{N})}^{N}\right)\mathrm{Pr}\left\{T^{N}=t^{N}\right\}\\ \;=\sum_{t^{N}:\left|ℑ\left(t^{N}\right)\right|<N\left(\alpha +\iota \right)}I\left(W;Y_{ℑ(t^{N})}^{N}\right)\mathrm{Pr}\left\{T^{N}=t^{N}\right\}\\ \;+\sum_{t^{N}:\left|ℑ\left(t^{N}\right)\right|\geq N\left(\alpha +\iota \right)}I\left(W;Y_{ℑ(t^{N})}^{N}\right)\mathrm{Pr}\left\{T^{N}=t^{N}\right\}\\ \;\overset{\left(c\right)}{\leq }\;\varepsilon^{\prime }+NC_{M}\mathrm{Pr}\{|ℑ\left(T^{N}\right)\left|\geq N\left(\alpha +\iota \right)\right\}\\ \;\;\overset{d}{\leq }\;\varepsilon^{\prime }\left(1+NC_{M}\right), \end{array}$$

where (a) follows because *W* is independent from $Z^{N}$; (b) follows because $Y_{I(t^{N})}^{N}$ is independent from $T^{N}$; (c) follows because $I\left(W;Y_{I(t^{N})}^{N}\right)\leq H\left(W\right)\leq NC_{M}$ when $|I\left(t^{N}\right)|>N\left(\alpha +\iota \right)$, and

$$I\left(W;Y_{I}^{N}\left(t^{N}\right)\right)\leq \max_{I\in {ℑ}_{\mu }}I\left(W;Y_{I}^{N}\right)<\varepsilon^{\prime }$$

when $|I\left(t^{N}\right)|<N\left(\alpha +\iota \right)$; and (d) follows from Formula (15). Consequently,

$$\frac{1}{N}I\left(W;Z^{N}\right)\leq \frac{\varepsilon^{\prime }}{N}\left(1+NC_{M}\right)<\varepsilon^{\prime }\left(1+C_{M}\right)<\varepsilon$$

when $\varepsilon^{\prime }$ is sufficiently small. The proof of the direct half is completed. □

## 6. Proof of Proposition 1

This section proves that $X^{n}\to Z^{n}\to T^{n}$ forms a Markov chain for every n∈N, which is Proposition 1. It suffices to prove that

(16) $$\begin{array}{l} Pr\left\{X^{n}=x^{n},Z^{n}=z^{n},T^{n}=t^{n}\right\}\mathrm{Pr}\left\{Z^{n}=z^{n}\right\}\\ \;=\mathrm{Pr}\left\{X^{n}=x^{n},Z^{n}=z^{n}\right\}\mathrm{Pr}\left\{Z^{n}=z^{n},T^{n}=t^{n}\right\} \end{array}$$

for any $x^{n}\in X^{n},t^{n}\in T^{n}$ and $z^{n}\in Z^{n}$. Suppose that $x^{n},t^{n}$ and $z^{n}$ are given. Denote

$$I\left(z^{n}\right)=\left\{1\leq i\leq n:z_{i}\neq ?\right\},$$

$$I\left(t^{n}\right)=\left\{1\leq i\leq n:t_{i}\in T_{1}\right\}.$$

If $I\left(z^{n}\right)\neq I\left(t^{n}\right)$, both sides of (16) equal 0. Formula (16) is established. If $I\left(z^{n}\right)=I\left(t^{n}\right)=I$, terms in Formula (16) are deduced as follows. Firstly,

(17) $$\begin{array}{l} Pr\{X^{n}=x^{n},Z^{n}=z^{n},T^{n}=t^{n}\}\\ \;=Pr\left\{X^{n}=x^{n}\right\}Pr\left\{T^{n}=t^{n}\right\}\cdot \\ Pr\{Z^{n}=z^{n}|X^{n}=x^{n},T^{n}=t^{n}\}\\ \;=Pr\left\{X^{n}=x^{n}\right\}Pr\left\{T^{n}=t^{n}\right\}\cdot \\ Pr\{Y_{ℑ}^{n}=z^{n}|X^{n}=x^{n},T^{n}=t^{n}\}\\ \;=Pr\left\{X^{n}=x^{n}\right\}Pr\left\{T^{n}=t^{n}\right\}\cdot \\ Pr\{Y_{ℑ}^{n}=z^{n}|X^{n}=x^{n}\}, \end{array}$$

where the last equality follows because $X^{n}$ and $Y^{n}$ are independent from $T^{n}$. Moreover,

(18) $$\begin{array}{l} Pr\{X^{n}=x^{n},Z^{n}=z^{n}\}\\ \;=Pr\{X^{n}=x^{n},Y_{ℑ}^{n}=z^{n}\}\\ \;=Pr\left\{X^{n}=x^{n}\right\}Pr\left\{Y_{ℑ}^{n}=z^{n}|X^{n}=x^{n}\right\}. \end{array}$$

Finally,

(19) $$\begin{array}{l} Pr\{Z^{n}=z^{n}T^{n}=t^{n}\}\\ \;=Pr\{Y_{ℑ}^{n}=z^{n},T^{n}=t^{n}\}\\ \;=Pr\left\{Y_{ℑ}^{n}=z^{n}\right\}Pr\left\{T^{n}=t^{n}\right\}, \end{array}$$

where the last equality follows because $Y^{n}$ is independent from $T^{n}$. Combining Formulas (17)–(19) results in Formula (16) also holding for $x^{n}$,$z^{n}$ and $t^{n}$ with $I\left(z^{n}\right)=I\left(t^{n}\right)$. The proof is completed.

## 7. Conclusions

Since the data in WBAN is highly related with the personal health, it is vital to protect this healthy information from attacks. In this paper, from the perspective of information theory, we studied the infrastructure of secure transmission system in WBAN, and solved the capacity problem of a class of finite-state Markov erasure wiretap channel for the IoT. The coding scheme used in this paper comes from the generalized wiretap channel II with the noisy main channel. The idea may be used to solve the capacity problems of other non-DMC wiretap channels. In a theoretical sense, the secure performance of our designed algorithm is not relevant with the computation capability of engaged computers and can guarantee the security of transmitted data in WBAN, by which the personal privacy could be significantly protected.

## References

[1] Tolossa Y.J., Vuppala S., Kaddoum G., Abreu G. (2017). On the uplink secrecy capacity analysis in D2D-enabled cellular network. IEEE Syst. J..

[2] Jameel F., Wyne S., Kaddoum G., Duong T.Q. (2018). A comprehensive survey on cooperative relaying and jamming strategies for physical layer security. IEEE Commun. Surv. Tutor..

[3] Kong L., Vuppala S., Kaddoum G. (2018). Secrecy Analysis of Random MIMO Wireless Networks over *α* − *μ* Fading Channels. IEEE Trans. Veh. Technol..

[4] Zhang P.N., Ma J. (2018). Channel Characteristic Aware Privacy Protection Mechanism in WBAN. Sensors.

[5] Anwar M., Abdyllah A.H., Butt R.A., Ashraf M.W., Qureshi K.N., Ullah F. (2018). Securing Data Communication in Wireless Body Area Networks Using Digital Signatures. Technol. J..

[6] Wyner A.D. (1975). The Wire-Tap Channel. Bell Syst. Technol. J..

[7] Kramer G. (2007). Topics in Multi-user Information Theory. Found. Trends Commun. Inf. Theory.

[8] Csiszar I., Korner J. (1978). Broadcast channels with confidential messages. IEEE Trans. Inf. Theory.

[9] Chen Y., Han Vinck A.J. (2008). Wiretap channel with side infor-mation. IEEE Trans. Inf. Theory.

[10] Dai B., Luo Y. (2012). Some new results on the wiretap channel with side information. Entropy.

[11] Dai B., Han Vinck A.J., Hong J., Luo Y., Zhuang Z. Degraded Broadcast Channel with Noncausal Side Information, Confidential Messages and Noiseless Feedback. Proceedings of the 2012 IEEE International Symposium on Information Theory.

[12] Dai B., Luo Y., Han Vinck A.J. Capacity region of broadcast channels with private message and causual side information. Proceedings of the 3rd International Conference on Image and Signal Processing (CISP 2010).

[13] Khisti A., Diggavi S.N., Womell G.W. (2011). Secrete-key agreement with channel state information at the transmitter. IEEE Trans. Inf. Forensics Secur..

[14] Chia Y.H., El Gamal A. (2012). Wiretap channel with causal state information. IEEE Trans. Inf. Theory.

[15] Ozarow L.H., Wyner A.D. (1984). Wire-tap channel II. AT T Bell Lab. Technol. J..

[16] He D., Luo Y. A kind of non-DMC erasure wiretap chan-nel. Proceedings of the 2012 IEEE 14th International Conference on Communication Technology.

[17] He D., Luo Y., Cai N. Strong Secrecy Capacity of the Wiretap Channel II with DMC Main Channel. Proceedings of the 2016 IEEE International Symposium on Information Theory (ISIT).

[18] Gilbert E.N. (1960). Capacity of a burst-noise channel. Bell Syst. Technol. J..

[19] Elliott E.O. (1960). Estimates of error rates for codes on burst-noise channels. Bell Syst. Technol. J..

[20] Wang H.S., Moayery N. (1995). Finite-state Markov channel—A useful model for radio communication channels. IEEE Trans. Veh. Technol..

[21] Lv N., Chen C., Qiu T., Sangaiah A.K. (2018). Deep Learning and Superpixel Feature Extraction based on Sparse Autoencoder for Change Detection in SAR Images. IEEE Trans. Ind. Inf..

[22] Chen C., Hu J., Qiu T., Atiquzzaman M., Ren Z. (2018). CVCG: Cooperative V2V-aided Transmission Scheme Based on Coalitional Game for Popular Content Distribution in Vehicular Ad-hoc Networks. IEEE Trans. Mob. Comput..

